# The Causes of HIV-Associated Cardiomyopathy: A Tale of Two Worlds

**DOI:** 10.1155/2016/8196560

**Published:** 2016-01-17

**Authors:** Rebecca H. Lumsden, Gerald S. Bloomfield

**Affiliations:** ^1^School of Medicine, University of Massachusetts Medical School, Worcester, MA 01605, USA; ^2^Department of Medicine, Duke Clinical Research Institute and Duke Global Health Institute, Duke University Medical Center, Durham, NC 27705, USA

## Abstract

Antiretroviral therapy (ART) has transformed the clinical profile of human immunodeficiency virus (HIV) from an acute infection with a high mortality into a treatable, chronic disease. As a result, the clinical sequelae of HIV infection are changing as patients live longer. HIV-associated cardiomyopathy (HIVAC) is a stage IV, HIV-defining illness and remains a significant cause of morbidity and mortality among HIV-infected individuals despite ART. Causes and clinical manifestations of HIVAC depend on the degree of host immunosuppression. Myocarditis from direct HIV toxicity, opportunistic infections, and nutritional deficiencies are implicated in causing HIVAC when HIV viral replication is unchecked, whereas cardiac autoimmunity, chronic inflammation, and ART cardiotoxicity contribute to HIVAC in individuals with suppressed viral loads. The initiation of ART has dramatically changed the clinical manifestation of HIVAC in high income countries from one of severe, left ventricular systolic dysfunction to a pattern of subclinical cardiac dysfunction characterized by abnormal diastolic function and strain. In low and middle income countries, however, HIVAC is the most common HIV-associated cardiovascular disease. Clear diagnostic and treatment guidelines for HIVAC are currently lacking but should be prioritized given the global burden of HIVAC.

## 1. Introduction

Dramatic gains have been made in the treatment of human immunodeficiency virus (HIV) over the last decade. By 2013, 35 million people globally were infected with HIV, and there were 2.1 million new HIV infections, nearly 40% lower than in 2001 [1]. The number of acquired immunodeficiency syndrome (AIDS) related deaths also declined by 35% over the same time period [1]. Much of the survival gains seen for people infected with HIV/AIDS are due to better availability of antiretroviral therapy (ART). The Joint United Nations Programme on HIV/AIDS (UNAIDS) estimates that 13.6 million people were receiving ART as of June 2014 and that 15 million will receive ART by 2015 [1]. HIV-infected individuals on ART can expect to live longer and, as a result, they are at risk of developing chronic, noncommunicable diseases including many forms of cardiovascular disease [2].

HIV-associated cardiomyopathy (HIVAC) has evolved since its first description in the mid-1980s [3]. Throughout the 1980s and 1990s, before the widespread availability of ART, the presence of heart failure in HIV-infected individuals was mainly in the context of myocarditis, related to direct effects of HIV, opportunistic infections, autoimmunity, nutritional deficiencies, or severe immunosuppression [4]. HIVAC was characterized as symptomatic, systolic dysfunction associated with a dilated left ventricle and indicated a poor prognosis for HIV-infected patients. Median survival was 101 days for HIV-infected patients after diagnosis with dilated cardiomyopathy, compared to 472 days for patients with normal findings on echocardiogram at a similar stage of immunosuppression [5]. Today, systolic dysfunction is being replaced by subclinical diastolic dysfunction as the hallmark of HIVAC in individuals with well controlled HIV [6].

No consensus criteria currently exist to define “HIV-associated cardiomyopathy,” but studies have outlined multiple subtypes of this evolving disease. Manifestations of HIVAC include symptomatic heart failure with left ventricular dysfunction with or without concurrent ventricular dilation, any systolic impairment or diastolic dysfunction in asymptomatic HIV patients, and new onset heart failure in stage IV HIV disease [7]. This broadened classification of HIVAC illustrates the increasingly complex relationship between HIV and cardiac dysfunction.

This transition in disease profile results from important disparities in the epidemiology and pathogenesis for HIVAC between high income countries (HICs) and low and middle income countries (LMICs), which, to the best of our knowledge, relate to differences in ART availability, HIV viral suppression, comorbidities, and opportunistic infections (Table 1) [6]. Thus, our understanding of the epidemiology and etiology of HIVAC in the pre-ART era remains relevant in many parts of the world where ART availability remains low. This review will explore the contributing etiologies of HIVAC while highlighting the current, disparate burden of HIVAC between HICs and LMICs.

## 2. Etiology of HIV-Associated Cardiomyopathy

Much of our understanding about the etiology of HIVAC is derived from studies performed in HICs before the availability of ART. As a result, the literature focused on direct and indirect cardiotoxicity of infections and HIV itself. More recent literature suggests an expanded role of autoimmunity and drug toxicity in the setting of ART. Studies from LMICs have also explored the role of nutrition in disease development. While large knowledge gaps remain, there are a number of prevailing hypotheses about the multifactorial etiology of HIVAC.

### 2.1. Myocarditis

Myocardial inflammation caused by HIV and related infections is implicated as a key inciting factor in the development of HIVAC. Various viral and opportunistic infections trigger myocarditis in the setting of uncontrolled HIV infection. Direct invasion of cardiac myocytes by cardiotropic viruses, including HIV, leads to a local cytokine release and subsequent infiltration of the myocardium with clonal expansion of B cells [8]. Myocarditis is particularly common in late stages of HIV infection. High rates of myocarditis are associated with CD4 counts of less than 400 cells/mm^3^ and up to two-thirds of untreated AIDS patients having histological evidence of myocarditis on autopsy [8, 9].

Both viral and nonviral opportunistic infections have been linked to myocarditis and subsequent left ventricular dysfunction in untreated HIV patients. One of the largest clinical pathology studies done to date found that Italian patients with AIDS and myocarditis were often coinfected with cardiotropic viruses, most commonly Coxsackie B3 virus (32%), Epstein-Barr virus (8%), and* Cytomegalovirus* (4%) [10, 11]. Even higher rates of* Cytomegalovirus* (48%) have been seen in patients with left ventricular dysfunction using in situ hybridization [12].* Toxoplasma gondii*,* Cryptococcus neoformans*, and* Mycobacterium avium-intracellulare* have also been isolated from the myocardium of end-stage AIDS patients with evidence of myocarditis and left ventricular dysfunction on autopsy [13]. Reduction in opportunistic infections in patients on ART may be responsible for the impressive drop in myocarditis rates and declining prevalence of HIVAC as seen in HICs [14, 15].

It is hypothesized that the HIV-1 virus causes myocarditis directly through myocyte toxicity, although debate about the exact pathogenesis exists. In vitro studies of human and rat cardiomyocytes have shown that HIV can enter myocytes directly through pathways independent of CCR5 and CXCR4 receptors. Invasion is thought to occur through macropinocytosis as HIV-1 virion particles with their nucleocapsid cores can be seen in vacuoles within myocytes on scanning electron microscopy [10, 16, 17]. HIV-1 nucleic acid sequences can be detected within the myocardial tissue of HIV-infected patients with myocarditis by in situ DNA hybridization [12, 18].

HIV also catalyzes a cascade of indirect pathways that induce myocardial inflammation and damage. Cardiomyocyte apoptosis and myocardial macrophage infiltration are more common in patients with HIVAC than in HIV-infected patients without cardiomyopathy [17]. Cardiomyocyte expression of HIV-1 associated protein, gp-120, and transactivator of transcription (Tat) protein signaling pathways have been implicated in mitochondrial dysfunction and cardiomyocyte apoptosis [16, 17, 19]. Additionally, myocardial dendritic cells including macrophages and endothelial cells have been considered “reservoir cells” for HIV-1 invasion and contribute to localized myocardial cell death through activation of inflammatory cytokines [20]. Macrophages initiate proapoptotic signaling through mitochondrial injury, activation of caspases, and receptor-mediated signaling through tumor necrosis factor- (TNF-) alpha and Fas ligand expression [16, 17]. The release of TNF-alpha specifically has been shown to have a negative ionotropic effect on cardiomyocytes by altering intracellular calcium homeostasis and inducing nitric oxide synthesis [10]. Myocardial damage from these indirect pathways ultimately leads to left ventricular systolic dysfunction, increased left ventricular mass, and expression of natriuretic peptides that may lead to hemodynamic compromise as demonstrated in vivo in rats [21].

### 2.2. Cardiac Autoimmunity

Higher levels of serum autoantibody titers have been seen in HIV-infected adults and children with myocardial disease compared to HIV-uninfected individuals. Significantly higher concentrations of anti-alpha myosin antibodies are found in HIV-infected individuals compared to HIV-negative controls [22]. The level of cardiac autoantibodies is progressively higher comparing HIV-uninfected controls (3%), HIV-infected individuals without heart disease (19%), and HIV-infected patients with left ventricular systolic dysfunction (43%) [22]. Autoantibody concentrations correlate with mortality in HIV-infected patients.

Infection with common and opportunistic viruses may facilitate the onset of cardiac autoimmunity in HIV-infected individuals by modifying cardiomyocyte surface antigens and exposing otherwise hidden cell surface epitopes, resulting in abnormal autoimmune responses against endogenous cardiomyocytes [22]. Persistent, latent myocardial infection with cardiotropic viruses, like* Cytomegalovirus*, may trigger clonal expansion of autoreactive CD8 T cells that target normal myocytes and lead to myocarditis [12].

### 2.3. Micronutrient Deficiency

Micronutrient deficiency is common in HIV-infected individuals due to gut malabsorption, diarrhea, and wasting syndrome. The resulting free radical formation and myocardial injury have been linked to the development of HIVAC. Selenium is the most widely studied micronutrient deficiency, as it plays a significant role in other forms of dilated cardiomyopathy. Selenium is an essential element used in the generation of glutathione peroxidase, an enzyme which protects lipid membranes from oxygen radicals and plays a crucial role in the prevention of myocardial injury [23]. Abnormalities in immunologic defense, phagocyte function, and T cell response, as seen with selenium deficiency, predispose to further myocardial injury [23]. Animal models have shown that selenium-deficient mice are more susceptible to myocyte damage and myocarditis when exposed to stressors, such as Coxsackie B virus [24].

Selenium deficiency has been associated with cardiomyopathy in untreated HIV-infected individuals. A prospective study of 416 HIV-infected patients in Rwanda found that low serum selenium levels were associated with nearly twice the odds of developing cardiomyopathy in multivariate analysis (OR 1.92, 95%CI 1.73–2.04) [25]. Low levels of serum selenium correlate directly with other known HIVAC risk factors, including low socioeconomic status and CD4 count [25, 26].

While selenium deficiency may have role in risk of HIVAC, the role of selenium supplementation in preventing or treating HIVAC remains unknown. Case reports have shown improvement in cardiac function with supplementation in targeted, selenium-deficient patients but, despite numerous salutary effects of selenium supplementation in HIV-infected individuals, no prospective evidence exists to support selenium supplementation for treating or preventing HIVAC [23, 27–29].

### 2.4. Antiretroviral Toxicity

In general the initiation of ART has decreased the prevalence of HIVAC in HIC, although use of zidovudine (AZT) based regimens may be associated with greater risk of cardiomyopathy [15, 27]. Zidovudine, a reverse nucleoside transcriptase inhibitor, inhibits mitochondrial DNA polymerase, causes mitochondrial damage, and leads to focal myocardial necrosis [30]. Treatment with AZT is associated with reversible, dose-dependent damage to skeletal and cardiac myocytes [30, 31]. Case reports of HIV-infected adults in the USA in the early 1990s revealed high rates of cardiac dysfunction associated with AZT monotherapy that rapidly reversed with cessation of AZT [32]. Increased left ventricular mass and peak wall stress have also been noted in HIV-infected children after treatment with AZT [33]. More recently, AZT exposure has also been linked to diastolic dysfunction in HIV-infected subjects [34].

### 2.5. Tuberculous Myopericarditis

Pericardial disease is often the first manifestation of cardiac disease in HIV-infected individuals and carries high mortality in LMICs [35]. Pericarditis caused by* Mycobacterium tuberculosis *(TB) is the leading cause of pericardial disease in HIV-infected individuals in highly endemic areas, accounting for up to 70% of all pericardial effusions and 90% of pericardial effusions in HIV-infected individuals in parts of Sub-Saharan Africa (SSA) [35, 36]. HIV infection is the most important predisposing factor for TB infection, and HIV infection is thought to alter the clinical manifestation of pericardial disease [36]. Direct pericardial invasion in HIV coinfection occurs through hematogenous spread of TB, unlike indirect lymphatic invasive in HIV-uninfected hosts [37]. In HIV-infected individuals, pericardial TB infection often results in larger pericardial effusions, more myopericardial involvement, and less constrictive pericarditis compared to HIV-uninfected individuals [36]. The Investigation of the Management of Pericarditis in Africa (IMPI Africa), a registry of 185 patients with suspected TB pericarditis from Cameroon, Nigeria, and South Africa, showed that patients with HIV were more likely to present with dyspnea and electrocardiographic changes, indicating myopericardial disease, and less likely to present with ascites, suggestive of a lower incidence of constrictive pericardial disease [38].

HIV coinfection with TB myopericarditis is a leading cause of cardiac death among HIV-infected patients, with a nearly sixfold increase in mortality compared to HIV-uninfected individuals [37]. HIV infection does not seem to alter the response to TB pericarditis treatment, although HIV-infected individuals have a higher rate of pericardial disease relapse [36]. The use of adjuvant corticosteroids to treat TB pericarditis in HIV-infected population remains controversial. A large, randomized controlled trial investigating the use of corticosteroids and/or* Mycobacterium indicus pranii* immunotherapy in TB pericarditis showed no difference between prednisolone and placebo or* M. indicus pranii* and placebo in the primary combined outcome of death, cardiac tamponade, and constrictive pericarditis, though patients receiving prednisolone as compared to placebo had significantly lower rates of the secondary outcomes of progression to constrictive pericarditis and fewer repeat hospitalizations [39]. There was also a significant increase in HIV-associated malignancy in HIV-infected patients receiving both prednisolone and* M. indicus pranii* versus placebo [39].

## 3. Effects of ART on Clinical Manifestations of HIVAC

The widespread use of ART has changed the phenotype of HIVAC as subclinical cardiac abnormalities, including diastolic dysfunction and impaired cardiac strain patterns, become increasingly common in HIV-infected individuals on effective HIV treatment [40]. A growing prevalence of asymptomatic ventricular dysfunction, abnormal strain patterns, and a higher incidence of diastolic dysfunction has been noted in HIV-infected populations on ART. The prevalence of systolic dysfunction has decreased in HICs whereas diastolic dysfunction is now seen in up to 64% of asymptomatic HIV-infected patients on ART [6, 40, 41]. Magnetic resonance imaging studies suggest that these subclinical changes may be due in part to myocardial fibrosis and steatosis seen in patients on ART [42]. While ART has dramatically reduced the burden of HIVAC in HICs, the incidence and mortality rates have risen in LMICs [14].

### 3.1. Burden of HIVAC in HICs

Most of our understanding of HIVAC emanates from studies performed in HICs, mostly from the United States and throughout Europe. Since the widespread initiation of ART the prevalence of HIVAC has dropped by 30% in these regions [14]. In the late 1980s, roughly one-third of all HIV-related cardiac deaths were due to dilated cardiomyopathy, and autopsy studies found evidence of myocarditis in up to 40% of noncardiac deaths in HIV-infected patients [35, 43]. A prospective study out of Johns Hopkins University in the early 1990s estimated the incidence of global left ventricular dysfunction to be 18% per year in HIV-infected patients [43]. However, with consistent access to antiretroviral medication and early initiation of treatment, myocarditis and dilated cardiomyopathy have virtually disappeared as manifestations of cardiac disease in HIV-infected patients in HICs today.

With the early advent of ART in HICs, the incidence of systolic dysfunction has decreased but diastolic abnormalities are increasing. A 2013 meta-analysis of 11 studies from HICs revealed that, among 2242 HIV-infected individuals on ART, only 8.3% had left ventricular systolic dysfunction whereas 43.4% had evidence of diastolic dysfunction [44]. Higher rates of subclinical cardiac abnormalities, such as abnormal left ventricular relaxation or pseudonormal filling patterns, higher pulmonary artery pressure, and decreased exercise tolerance are more frequently observed in patients on ART [40].

Additionally, the burden of cardiac disease in HIV infection in HICs are transitioning towards increasing atherosclerosis and ischemic heart disease. Patients on ART in HICs are living longer and exposed to more traditional cardiac risk factors such as tobacco use, hyperlipidemia, and diabetes. Antiretroviral therapies have been linked to an increased risk of coronary artery disease and myocardial infarction as well as acceleration of atherosclerotic formation and metabolic disturbances. Generally, immune reactivation with ART and chronic low-grade inflammation have been shown to promote subclinical atherosclerotic changes and arterial stiffness [45, 46]. The three major classes of ART, protease inhibitors (PIs), nucleoside reverse transcriptase inhibitors (NRTIs), and nonnucleoside reverse transcriptase inhibitors (NNRTIs), have all been associated with some degree of dyslipidemia; PIs and the NRTIs, stavudine and zidovudine, are indirectly implicated in the development of atherosclerosis via significant alterations in lipid metabolism and insulin resistance [45, 47, 48]. The Data Collection on Adverse Events of Anti-HIV Drugs (D:A:D) study, an international collaboration representing over 30,000 HIV-infected patients across Europe, the United States, and Australia, found an increased risk of myocardial infarction with use of PIs and certain NRTIs, namely, abacavir and didanosine [45, 49–51]. However, a 2013 systematic review of 27 studies addressing the risk of cardiovascular disease from ART did not find a consistent relationship between these drugs and myocardial risk [52]. Further prospective, randomized controlled trials are needed to better assess the relationship between ART and myocardial infarction risk. Meanwhile, the long term benefits of ART on controlling HIV infection and disease sequelae are thought to outweigh the increased relative risk of cardiovascular disease in the HIV-infected population.

### 3.2. Burden of HIVAC in LMICs

The impact of HIVAC may be most severe in LMICs, where HIVAC remains a relevant cause of morbidity and mortality despite the expanding use of ART [4]. HIVAC is associated with low socioeconomic status, a longer duration of HIV infection, low total lymphocyte count, low CD4 count, high HIV-1 viral load, and low plasma levels of selenium [37]. A CD4 count <100 cells/mm^3^ appears to be an important threshold below which the risk of developing HIVAC increases significantly [5]. Cross-sectional studies in the pre-ART era from SSA indicated a prevalence of cardiomyopathy in up to 57% in hospitalized patients [35]. A prospective study of 157 HIV- infected patients in Kinshasa, Democratic Republic of Congo, showed that about half of the patients developed a cardiac abnormality over 7 years [13].

More recently, the Heart of Soweto study found that, of the 5328 newly diagnosed cases of cardiac disease at a major hospital in South Africa between 2006 and 2008, 518 cases were in HIV-infected patients and only half of them (54%) were taking ART [7]. The most common cardiac diagnosis among all HIV-infected patients was HIVAC (38%) with an average left ventricular ejection fraction of 46%. The viral loads were significantly higher (110,000 versus 90,000 RNA copies/mL) and CD4 counts significantly lower (180 versus 211 cells/mm3) in cases of HIVAC compared to those HIV-infected patients without cardiomyopathy [7]. Results from the Sub-Saharan Africa Survey of Heart Failure study, a multinational registry of patients across Africa presenting to hospitals with acute, decompensated heart failure, showed that HIV was the direct cause of heart failure in 2.6% of all cases [53].

The mortality due to HIVAC is significant and reaches as high as 15–20% in parts of SSA [54]. HIV status is an independent predictor of death at 180 days for patients with acute decompensated heart failure and is associated with increased in-hospital, 60-day, and 180-day mortality rates [55].

### 3.3. Reconciling HIVAC Disparities in HICs and LMICs

The causes contributing to HIVAC seem to depend on the degree of viral suppression which are strongly related to region of the world [35]. Opportunistic and viral infections, nutritional deficiencies, and direct HIV toxicity are leading causes in uncontrolled disease, especially with high viral loads or CD4 counts <100 [4]. When viral suppression is adequate and immune function is restored, ART, chronic inflammation, and autoimmunity may be more pronounced contributors to HIVAC [4]. Thus, HIVAC may truly represent yet another syndrome of heart failure with numerous individual causes, each of which may warrant specific therapy in addition to generally accepted therapy for heart failure. As life expectancy for HIV-infected individuals continues to increase worldwide, we are likely to see more subclinical manifestations of HIVAC which warrant more attention to screening in the presymptomatic individual.

## 4. Current Diagnostic and Screening Tools

 Identifying early markers of myocardial dysfunction in HIV-infected individuals at high risk of cardiac disease may provide early intervention of life-saving therapy. To date, however, there have been no diagnostic criteria or screening guidelines defined for HIVAC. Echocardiography remains the standard for detection of ventricular dysfunction [56]. Diastolic dysfunction and abnormal myocardial strain are often the only echocardiographic abnormalities in asymptomatic HIV-infected patients on ART [4]. Early detection of subclinical myocardial dysfunction can be assessed by 2-dimensional strain and strain rate using speckle tracking echocardiography [37]. Further, cardiac magnetic resonance can now detect signs of subclinical cardiac steatosis and myocardial fibrosis [42]. However, the clinical significance of some of these structural and metabolic cardiac changes remains unknown.

The role of screening echocardiography in HIV-infected populations is unclear. Timing and frequency of echocardiography testing is undetermined. Starc et al. have recommended that pediatric patients have an echocardiogram done at the time of HIV-diagnosis, followed by repeat testing every couple of years for asymptomatic patients or annual testing in patients with symptoms of heart failure, unexplained respiratory illness, or symptomatic HIV infection [57]. Given the increasing prevalence of subclinical disease and poor outcomes in late detection of systolic dysfunction in HIV-infected patients, developing clear screening guidelines should be a high priority. However, even with optimized screening practices and diagnostic criteria, targeted treatment options remain limited once HIVAC develops.

## 5. Current Treatment Options

Best practices for treatment of HIVAC have not been rigorously tested. Early initiation of beta-blockers and ACE-inhibitor therapy may be beneficial in subclinical disease to prevent progression to severe systolic dysfunction through common mechanisms, such as afterload reduction and sympathoadrenal modulation [56, 58]. In the absence of specific guidelines to the contrary, patients with HIV and heart failure should be treated with standard therapy for heart failure according to current consensus guidelines [59].

ART has been shown to positively impact outcomes in retrospective studies, but there is no prospective evidence that ART has a beneficial effect on cardiac outcomes in HIVAC [20]. Such evidence is unlikely to be forthcoming, however, as the latest WHO guidelines recommend initiating ART regardless of CD4 count. Adjunctive therapies for HIVAC such as supplementation with carnitine, selenium, and multivitamins have been proposed in an attempt to preserve left ventricular function in micronutrient deficient populations but warrant further evidence before wide scale adoption [60]. Immunomodulatory therapy has been shown to improve left ventricular structure and function in some patient populations. Patients with biopsy-proven autoimmune myocarditis, for example, improve left ventricular function and dimensions after therapy with corticosteroids [61]. Monthly intravenous immunoglobulin (IVIG) infusions have also been shown to improve cardiac function in HIV-infected children with subclinical cardiac abnormalities [20, 62]. However, there are no controlled trials investigating efficacy of corticosteroids or IVIG in treating HIVAC in adult populations. Further investigation is needed to identify best treatment practices for HIVAC.

Mechanical support devices and cardiac transplantation are definitive treatment options for end-stage HIVAC, although their use is still limited in HIV-infected populations. HIV infection was previously considered a contraindication to mechanical support and transplant, but since advanced ART has improved outcomes and mortality rates from end-stage heart failure continue to rise, the United Network for Organ Sharing (UNOS) declared that asymptomatic HIV-infected individuals should not be excluded from heart transplant consideration solely based on their HIV status [63]. Data from case series and small cohort studies in the USA and Canada suggest that good outcomes with survival rates for HIV-infected patients are similar to those of HIV-uninfected patients up to 3 years after cardiac transplantation [64, 65]. Despite this evidence, a recent survey of cardiac transplantation centers found that 57% of programmes still considered HIV infection to be a contraindication to transplantation due to scarcity of organ supply, concerns for posttransplant immunosuppression enhancing progression to AIDS, and possible postoperative drug interactions between ART and immunosuppressive therapies [66]. Left ventricular assist devices (LVADs) are also scarcely used in HIV-infected individuals, with most centers citing risks of device-related infection [66]. A case study of two HIV-infected individuals who underwent implantation with HeartMate XVE pulsatile-flow LVAD found no HIV-related infectious complications, and a recent analysis of all 22 HIV-infected LVAD cases in the USA revealed outcomes similar to the general LVAD population with comparable mortality rates at 3, 6, 12, and 24 months [66, 67].

## 6. Biomarkers for HIVAC Screening

The use of novel biomarker testing to screen for cardiac dysfunction in HIV-infected persons is a growing area of investigation. B-natriuretic peptide (BNP) screening combined with collaborative care has been shown to reduce the rates of systolic and diastolic dysfunction in patients at risk of heart failure [68]. An inverse correlation between BNP levels and left ventricular function in HIV-infected patients has been seen in small case studies [69, 70], but the specificity of BNP for cardiac disease in HIV-infected individuals is unclear [69–71]. More research is needed to assess whether this cost-effective and simple test may be a useful screening tool for identifying HIVAC.

Soluble ST2, a novel biomarker of cardiac stress, and GDF-15, a growth differentiation factor expressed in cardiac injury, are associated with cardiac dysfunction and all-cause mortality in a controlled study of HIV-infected individuals [72]. ST2 was also associated with diastolic dysfunction, suggesting its role as a possible profibrotic mediator in HIVAC. Other novel markers requiring further investigation include serum autoantibody titers for cardiac-specific autoantibodies, like anti-alpha myosin, that have been identified in left ventricular dysfunction in HIV-infected individuals and may serve as a target for immunomodulatory treatment [10].

## 7. Conclusion

HIV-associated cardiomyopathy remains a significant cause of morbidity and mortality in both HICs and LMICs despite the widespread use of ART. Overall, the clinical presentation of HIVAC is changing as life expectancy increases in HIV-infected individuals. Severe, symptomatic dilated cardiomyopathy, as previously seen in end-stage AIDS, is declining as the predominant clinical manifestation of HIVAC. Subclinical, diastolic dysfunction and abnormal ventricular strain patterns are being seen more frequently in HIV-infected individuals with adequate HIV viral control. The etiology for this variable phenotype likely depends on the degree of viral replication and immunosuppression. Myocarditis, opportunistic infections, micronutrient deficiencies, and HIV itself play a large role in individuals with inadequate viral suppression and poor immune function, whereas ART toxicity and cardiac autoimmunity are seen more when disease is controlled.

The prevalence of HIVAC has declined in HICs with successful ART and decreased opportunistic infections, whereas HIVAC remains a significant contributor to disease burden in LMICs [7, 14, 27]. These diverging epidemics result from a combination of factors. Poor soil composition across SSA has predisposed a quarter of the population to selenium and other micronutrient deficiencies that have been seen to worsen cardiomyopathy [4]. Limited access to effective ART is a critical challenge faced in many LMICs. Frequent use of AZT in first-line therapy persists in many LMICs due to its low cost despite international recommendations for other, less cardiotoxic regimens [73].

Emphasis needs to be placed on designing clear guidelines for screening protocols and diagnostic criteria for HIVAC. Appropriate timing and tools for cardiac screening in HIV-infected individuals beg clarification. Using advanced echocardiographic imaging to evaluate for contractile reserve, diastolic dysfunction, and abnormalities in myocardial deformation can identify higher-risk patients [4], but it remains unknown whether this alters clinical decision making for HIV-infected patients. Establishing diagnostic criteria that account for stage of HIV and degree of immunosuppression should be a high priority. Recommendations regarding the timing and frequency of routine cardiac evaluation for HIV-infected individuals are needed, as well as partnership between infectious disease specialists and cardiologists in identifying and managing patients at high risk of HIVAC. HIV-associated cardiomyopathy will continue to be a significant contributor to the global cardiac disease burden as the HIV population ages, and more research is needed to understand best practices in diagnosis and treating the disease worldwide.

## Figures and Tables

**Table 1:** Etiologies and Characteristic Phenotypes of HIVAC.

	Etiology of HIVAC	Characteristic HIVAC Phenotype
*Uncontrolled HIV Disease*: (i) Immunosuppressed host (ii) High viral load (iii) Low CD4 count (<400 cells/mm^3^)	(i) Myocarditis (a) Direct HIV toxicity (b) Opportunistic Infections (1) Viral: Coxsackie B, CMV, EBV (2) Non-viral: Toxoplasmosis, Cryptococcus, MAC (ii) Tuberculous Myopericarditis (iii) Micronutrient Deficiency (a) Selenium Deficiency	(i) More commonly seen in LMIC (ii) Symptomatic, systolic dysfunction +/− dilated ventricles (iii) Poor prognosis

*Controlled HIV Disease*:	(i) Cardiac Autoimmunity	(i) More commonly seen in HIC
(i) Immunocompetent host	(ii) Cardiac inflammation	(ii) Subclinical diastolic dysfunction with increased strain patterns
(ii) Undetectable viral load	(iii) ART toxicity	
	(a) AZT-induced cardiomyopathy	
